# Bias–selectable nBn dual–band long–/very long–wavelength infrared photodetectors based on InAs/InAs_1−x_Sb_x_/AlAs_1−x_Sb_x_ type–II superlattices

**DOI:** 10.1038/s41598-017-03238-2

**Published:** 2017-06-13

**Authors:** Abbas Haddadi, Arash Dehzangi, Romain Chevallier, Sourav Adhikary, Manijeh Razeghi

**Affiliations:** Center for Quantum Devices, Department of Electrical Engineering and Computer Science, Northwestern University, Evanston, IL 60208 USA

## Abstract

Type–II superlattices (T2SLs) are a class of artificial semiconductors that have demonstrated themselves as a viable candidate to compete with the state–of–the–art mercury–cadmium–telluride material system in the field of infrared detection and imaging. Within type–II superlattices, InAs/InAs_1−x_Sb_x_ T2SLs have been shown to have a significantly longer minority carrier lifetime. However, demonstration of high–performance dual–band photodetectors based on InAs/InAs_1−x_Sb_x_ T2SLs in the long and very long wavelength infrared (LWIR & VLWIR) regimes remains challenging. We report the demonstration of high–performance bias–selectable dual–band long–wavelength infrared photodetectors based on new InAs/InAs_1−x_Sb_x_/AlAs_1−x_Sb_x_ type–II superlattice design. Our design uses two different bandgap absorption regions separated by an electron barrier that blocks the transport of majority carriers to reduce the dark current density of the device. As the applied bias is varied, the device exhibits well–defined cut–off wavelengths of either ∼8.7 or ∼12.5 μm at 77 K. This bias–selectable dual–band photodetector is compact, with no moving parts, and will open new opportunities for multi–spectral LWIR and VLWIR imaging and detection.

## Introduction

Over the course of the last decade, infrared imaging has seen tremendous improvements thanks to the introduction of new technologies. However, no longer are single–waveband photodetectors sensitive enough for all applications and the ability to see the objects in multiple wavebands through a single infrared camera can be indispensable. Multi–spectral infrared imagers offer a number of advantages by adding spectral selectivity. The comparison of colored photography and television compared to black/white is the best example of the motivation to have two or multi–spectral detection. Colored pictures and video do not just give better–looking images, but can also be used to extract additional useful information. Each additional collected waveband increases the information capacity for image processing algorithms and can enhance the fidelity of the scene. This can result in enhanced capabilities to identify and track targets from among the background clutter. Moreover, multi–spectral infrared imaging covering separate atmospheric windows can offer better discrimination due to the higher signal contrast seen between wavebands from the targets’ chemical signature and/or thermal emission^1^.

Long–wavelength infrared (LWIR) photodetectors are, particularly, suitable for terrestrial–based infrared imaging since the emission peaks of room temperature objects are po = sitioned in the 8 to 12 μm atmospheric window according to Planck’s blackbody law. InAs/GaSb/AlSb Type–II superlattices (T2SLs) have emerged as a highly suitable candidate for multi–spectral detection due to the ease with which the bandgap can be tuned, while retaining closely lattice–matched conditions^1–4^. As a result, high quantum efficiency (QE) focal plane array cameras have been demonstrated^5^, comparable to other state–of–the–art technologies such as mercury–cadmium–telluride (HgCdTe) compounds. In addition, because T2SLs are a III–V semiconductor material system, the production of T2SL–based imagers has the potential to allow leverage of existing commercial III–V foundries to further drive down costs^6^. Another T2SL advantage is its spectral spatial uniformity already proven in the LWIR band^7^, which is a critical element for the yield of larger high–resolution imagers.

InAs/InAs_1−x_Sb_x_ T2SLs are the newest members of T2SLs family, and they have been shown to have significantly longer minority carrier lifetimes than more conventional InAs/GaSb/AlSb T2SLs^8–10^ and are actively being pursued as an alternate to InAs/GaSb/AlSb T2SLs–based infrared photodetectors and imagers^11–14^. Even though sharing the same type–II band alignment, these two types of T2SLs witness differences in superlattice design. GaSb, AlSb, and InAs belong to the 6.1 A family. Therefore, not only does the variation of layer thicknesses in superlattice design enjoy a large scale of freedom, there are also a variety of novel hetero–structures possible, such as the W–structure^15^ or M–structure^16^ for pMp^17^ or nBn^18^ device architectures. On the contrary, InAs_1−x_Sb_x_ and InAs can have very different lattice constants; thus, the constituent layer thicknesses and the antimony molar composition (x) in the InAs_1−x_Sb_x_ layer should be taken into account to control the strain. This is, especially, true when going to the LWIR and VLWIR regime in which a large fraction of antimony in the InAs_1−x_Sb_x_ layers is required^11^. Facing these constraints, the device designers should address more challenges in the InAs/InAs_1−x_Sb_x_ T2SLs material system especially when it comes to thick dual–band LWIR photodetectors.

We have developed a novel variant of the InAs/InAs_1−x_Sb_x_ T2SLs that incorporates additional AlAs_1−x_Sb_x_ layers into the superlattice. These extra layers allow additional freedom in designing the device and allow us to demonstrate high–performance dual–band photodetectors that can perform sequentially as two individual single–band photodetectors according to the polarity of the applied bias voltage.

## Results

### Bias–selectable dual–band long wavelength photodetector design

**Figure 1:**
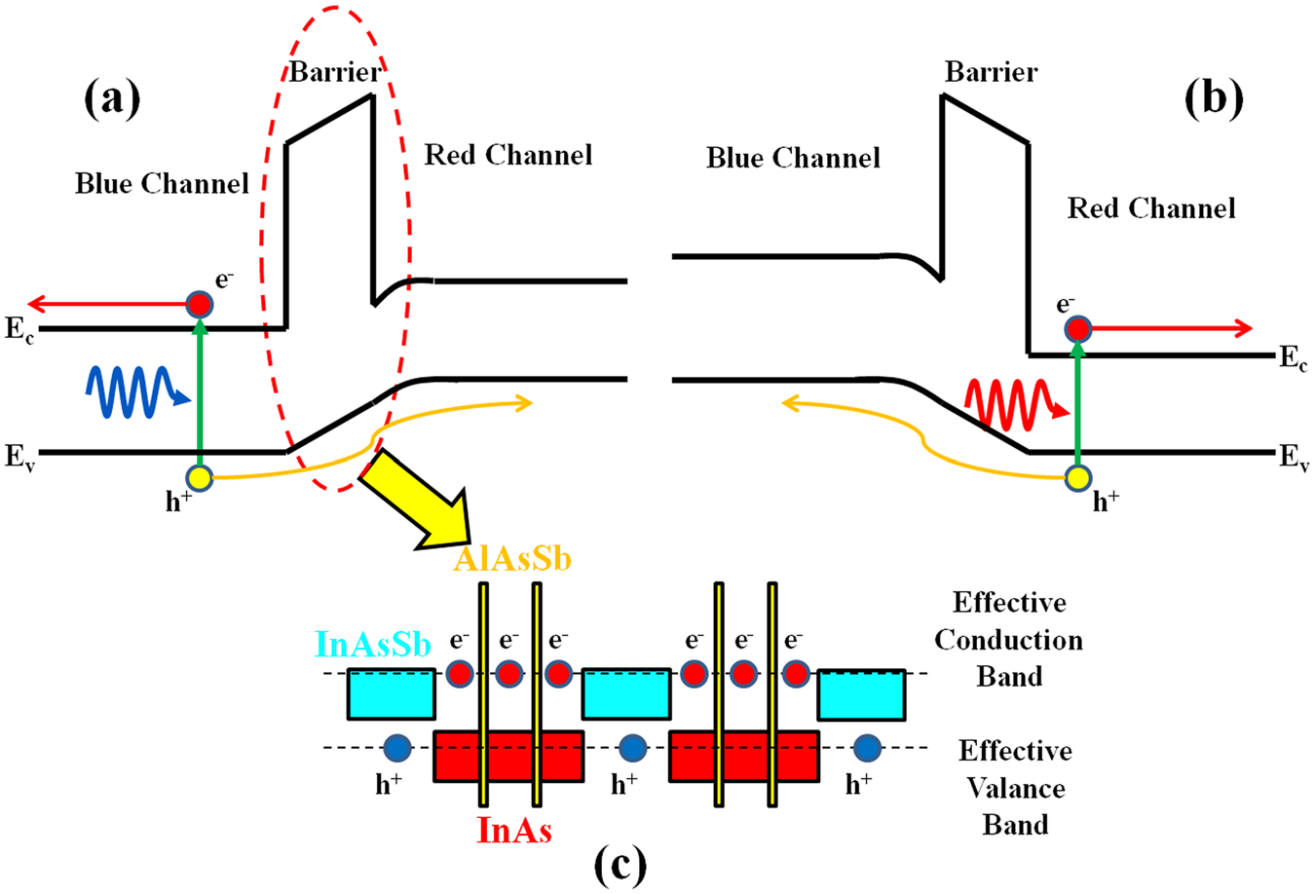
The schematic diagram and working principle of the bias-selectable nBn dual-band LWIR/VLWIR photodetector under (**a**) positive, and (**b**) negative applied bias voltage. The barrier blocks the transport of majority electrons, while, only, allowing the transport of minority holes and photo-generated carriers. (**c**) The band alignment and the creation of an effective bandgap in saw-tooth InAs/AlAs_1−x_Sb_x_/InAs/AlAs_1−x_Sb_x_/InAs/InAs_1−x_Sb_x_ superlattice of the barrier. Colored rectangles represent the forbidden band gap of the materials. The atomic engineering capabilities of T2SLs enable near–perfect valence band alignment of all three regions.

We used a modified nBn^18^ photodetector architecture that consists of a *n*–doped InAs/InAs_1−x_Sb_x_ T2SL–based absorption region for the red channel, a *n*–doped InAs/AlAs_1−x_Sb_x_ /InAs_1−x_Sb_x_ T2SL–based absorption region for the blue channel, and a thin InAs/AlAs_1−x_Sb_x_ /InAs_1−x_Sb_x_ T2SL–based electron–barrier which is sandwiched between the two absorption layers. The barrier superlattice design is chosen so that the electron–barrier has zero valence band discontinuity with respect to both *n*–type absorption regions, which allows transport of minority carriers (holes) while blocking transport of majority carriers (electrons). An external electric field is then applied to the device to introduce a band–bending in the valence band while the electron–barrier continues to block the conduction band. The polarity of this applied bias determines from which channel the photo–generated carriers are extracted. Figure 1(a) and (b) sketch the schematic diagram and working principle of this nBn dual–band photodetector under small positive and negative applied biases, respectively. The device does not need an additional middle contact which makes the photodetector fabrication process simpler and keeps each channel quite independent of each other.

Prior to demonstrating of bias–selectable dual–band LWIR nBn photodetectors, one has to overcome two major challenges simultaneously. The first challenge is to design proper superlattices for both absorption regions and the electron–barrier that are all lattice–matched to GaSb substrate; the second one is to adjust the superlattice designs to have zero valance band discontinuity.

The design process begins with choosing superlattice designs with ∼8 and ∼12 μm cut–off wavelengths and similar valence band energy level to minimize the bias dependency of the device. At the same time, the superlattice designs have to take into consideration the strain balance to ensure high–quality material, which is necessary for a long minority carrier diffusion length and high quantum efficiency (QE). The superlattice design of the red absorption region consists of 30/10 mono–layers (MLs) of InAs/InAs_0.48_Sb_0.52_, respectively, per period with a ∼12 μm nominal cut–off wavelength at 77 K. The superlattice design of the blue absorption region consists of 13/1.5/13/9 MLs of InAs/AlAs/InAs/InAs_0.48_Sb_0.52_, respectively, per period with an ∼8 μm nominal cut–off wavelength at 77 K. The additional AlAs layer in the blue channel superlattice provides the necessary flexibility in the bandstructure design and helps compensate the excessive tensile strain generated by the InAs_1−x_Sb_x_ layer of the superlattice.

Designing the electron–barrier is the final critical step of the device design process. Since holes are localized in the InAs_1−x_Sb_x_ layers, and thus the valence band level is directly related to its composition and thickness, we intentionally tried keep the InAs_1−x_Sb_x_ layer thickness in the barrier close to the InAs_1−x_Sb_x_ layer thicknesses in the blue and red absorption regions. The electron barrier design consists of 4/3/4/3/4/9 MLs of InAs/AlAs_0.55_Sb_0.45_/InAs/AlAs_0.45_Sb_0.55_/InAs/InAs_0.45_Sb_0.55_, respectively, per period with a nominal cut–off wavelength of ∼4 μm at 77 K. Because AlAs_0.45_Sb_0.55_ has a lower lattice mismatch to the GaSb substrate^14^ compared to AlAs^11^, using it in this barrier design provides more flexibility to accommodate the lattice strain and allows the superlattice to be grown with thicker large–bandgap aluminum–content layers. Furthermore, inserting two spatially–separated AlAs_0.45_Sb_0.55_ high–bandgap layers inside the InAs quantum well (see Fig. 1c) results in a higher conduction band energy level while maintaining high crystalline quality. This structure is called a saw–tooth superlattice, and can provide a highly controllable electron–barrier to effectively block the transport of the majority electrons in the conduction band while ensuring zero valence band discontinuity with both blue and red absorption regions and allowing the free movement of minority holes.

**Figure 2:**
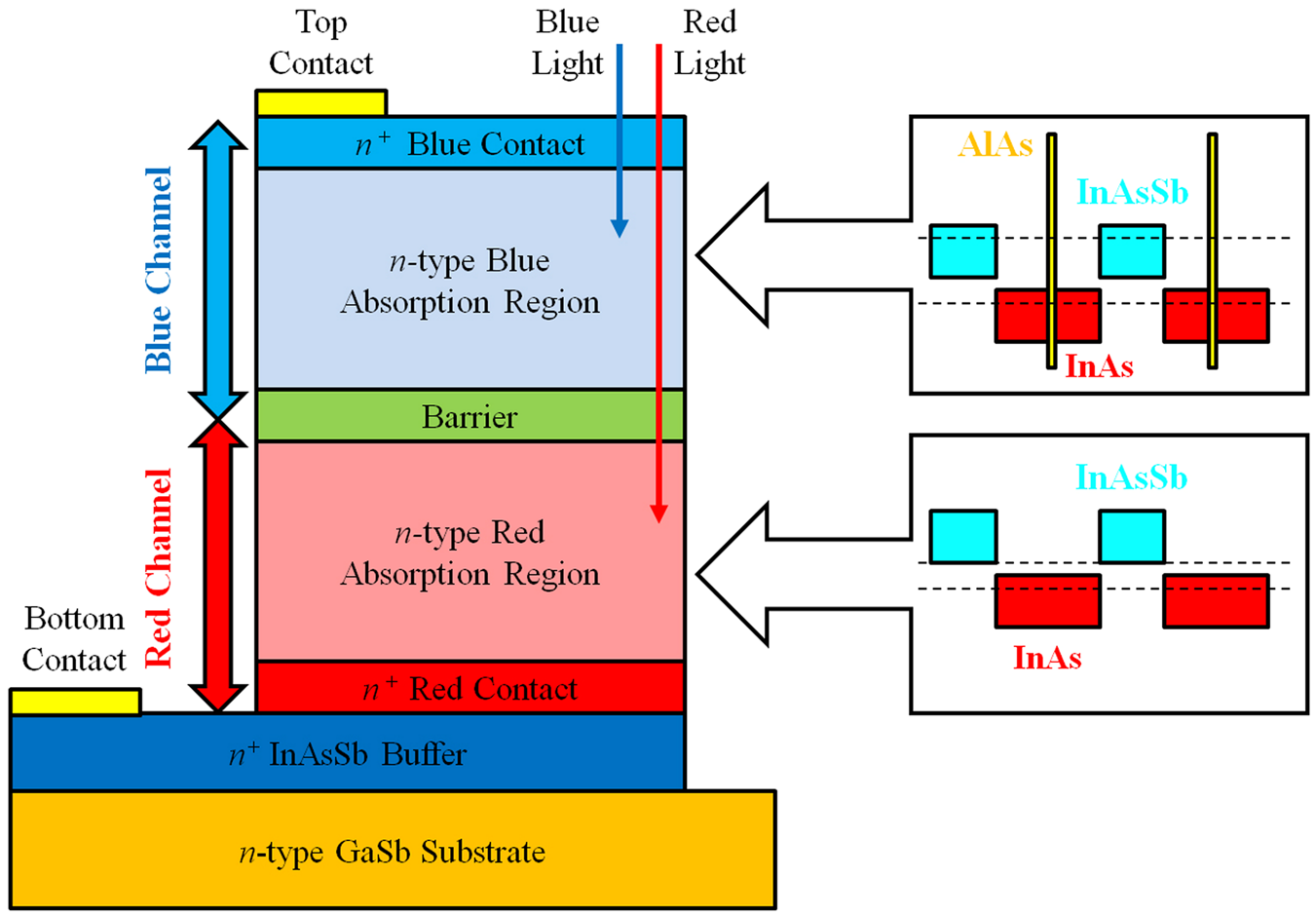
The schematic diagram of the bias-selectable dual-band nBn LWIR/VLWIR photodetector structure and the schematic band alignment of superlattices in both the blue and red absorption regions. The colored rectangles in the insets represent the forbidden gap of the constituent layers. The dashed lines represent the effective bandgaps of the superlattices.

During device epitaxial growth, the blue channel was grown on top of the red channel in order to allow response from both channels in the front–side illumination configuration. The blue channel plays a role as a low–pass photon energy filter for the bottom red channel in this device configuration (see Fig. 2).

Following the device design process, the material was grown on a Te–doped *n*–type (10^17^ cm^−3^) GaSb substrate using a solid–source molecular beam epitaxy (SSMBE) reactor. The growth was nucleated with a ∼100 nm GaSb buffer layer; then, a 0.5 μm *n*–doped InAs_0.91_Sb_0.09_ buffer layer (10^18^ cm^−3^) was grown, followed by a 0.0035 μm–thick red *n*–contact (10^18^ cm^−3^), a 4 μm–thick *n*–type red absorption region (10^16^ cm^−3^), a 0.5 μm–thick saw–tooth superlattice–based electron barrier, a 4 μm–thick *n*–type blue absorption region (10^16^ cm^−3^), and finally a 0.5 μm–thick blue *n*–contact (10^18^ cm^−3^). The blue and red *n*–contacts’ superlattice designs are similar to the blue and red absorption regions’ superlattice designs, respectively. Silicon (Si) was used as the *n*–dopant to facilitate formation of ohmic contacts.

**Figure 3:**
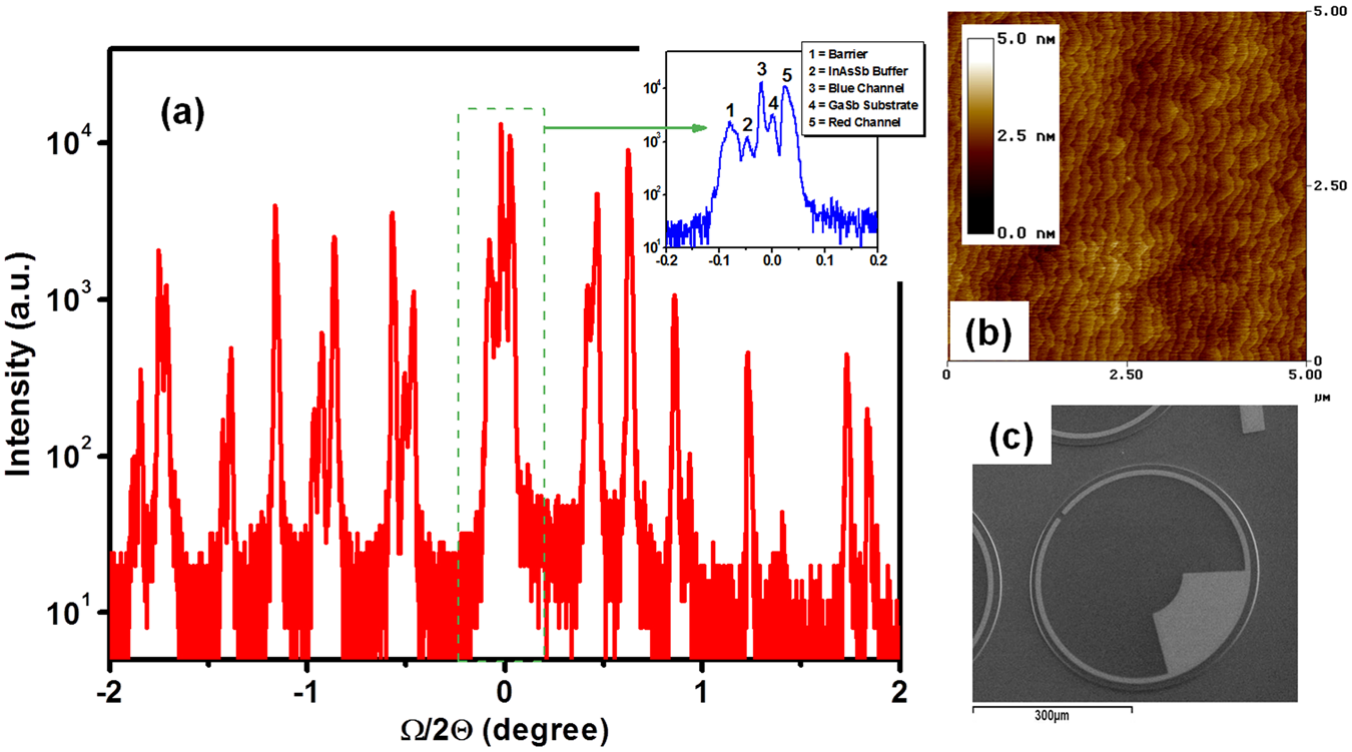
(**a**) High–resolution X–ray diffraction of the material grown for the device (inset: detail view showing just the zero–order peaks of the superlattices, InAsSb buffer layer, and GaSb substrate). (**b**) The atomic force microscopy of a 5 × 5 μm^2^ surface area of the material grown for the device with an RMS roughness value of 1.18 Å. (**c**) A scanning electron microscopy image of a processed single-element device.

After epitaxial growth, the material quality was assessed using atomic force microscopy and high–resolution X–ray diffraction (HR–XRD). The satellite peaks in the HR–XRD scan show the overall periods of the red absorption region, the blue absorption region, and the electron barrier were about 125, 111, and 82 Å, respectively (Fig. 3a). Both absorption regions and the barrier are lattice–matched to the GaSb within 1500 ppm, as was expected. The atomic force microscopy shows a good surface morphology with clear atomic steps and a root mean squared (RMS) roughness of 1.18 Å over a 5 × 5 μm^2^ area (see Fig. 3b).

After structural quality assessment, the material was processed into a set of unpassivated circular and square mesa–isolated test structures with device sizes ranging from 100 × 100 to 400 × 400 μm^2^ ^19^. Figure 3(c) shows a scanning electron microscopy image of a processed circular photodetector. The photodetectors were left unpassivated; however, special attention was paid in order to minimize the surface leakage current. Finally, the sample was wire–bonded to a 68 pin leadless ceramic chip carrier (LCCC) and loaded into a cryostat for both optical and electrical characterization at 77 K.

### Dual–band photodetector electro–optical characterization results

**Figure 4:**
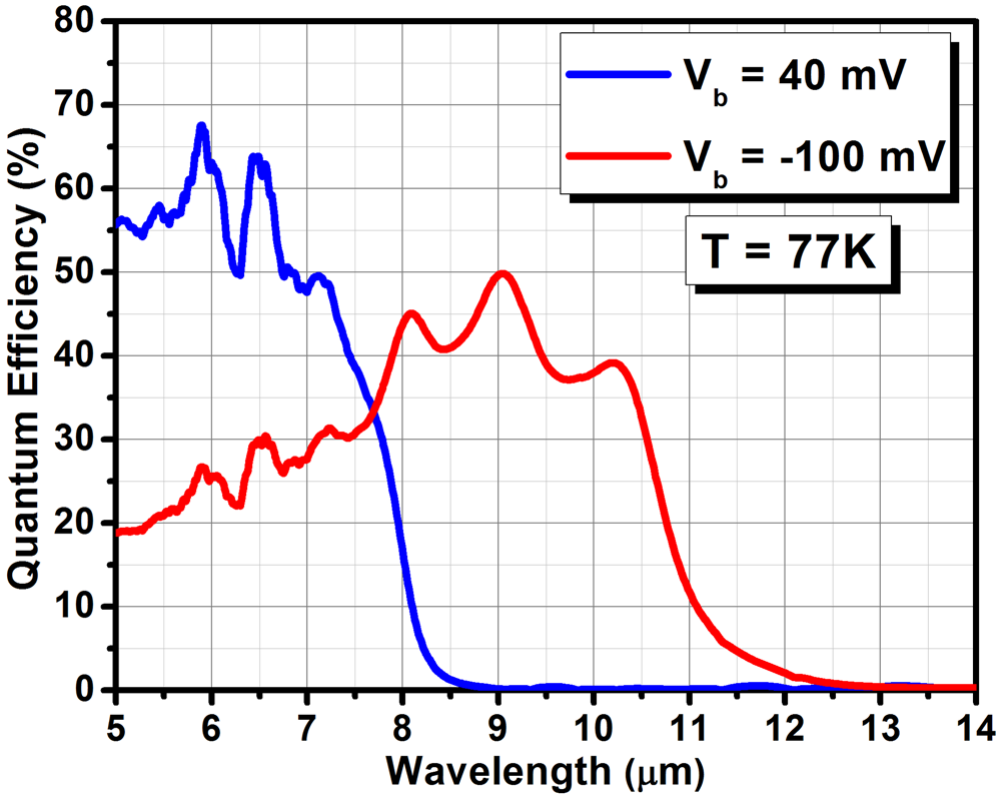
Quantum efficiency spectrum of the photodetector at in front-side illumination configuration without any anti-reflection coating at 77 K. The devices acts like a LWIR (blue) photodetector at +40 mV applied bias and a VLWIR (red) photodetector −100 mV bias voltage.

The optical characterization of the photodetectors was done at 77 K under front–side illumination. No anti–reflection (AR) coating was applied to the photodetectors. Applied biases ranging from positive down to −20 mV corresponds to the transport of minority holes collected from the blue absorption region and lead to LWIR detection, while larger negative biases (below −20 mV) correspond to the transport of holes collected in the red absorption region and lead to VLWIR detection. The blue and red optical response saturate at +40 and −100 mV, respectively. Figure 4 shows the saturated optical performance of each absorption region. The blue and red channels exhibit 100% cut–off wavelengths of ∼8.6 μm (∼145 meV) and ∼12.5 μm (∼100 meV) at 77 K, respectively. The blue channel exhibits a saturated QE of 65% at 6.45 μm and the red channel exhibits a saturated QE of 50% at 9 μm. This corresponds to saturated peak responsivities of 3.37 A/W at 6.45 μm for the blue channel and 3.7 A/W at 9 μm for the red channel. The performance of both channels saturates at relatively low bias voltages which makes this device well suited for FPA applications. The small bias dependency of the QE spectrum also suggests that a good valence band continuity has been achieved in this device. The optical performance of both channels are comparable to the best reported values for more conventional InAs/GaSb T2SL–based bias–selectable dual–band LWIR/VLWIR photodetectors^12^.

In the dual–band LWIR/VLWIR photodetectors with broad-band absorption regions (such as our dual–band photodetector), incomplete absorption of LWIR (λ < 8 μm) in the blue absorber results in absorption of LWIR in the red absorber (see Fig. 4). This leads to a response from the red channel (negative bias operation) to LWIR light, which is referred to as spectral cross–talk (SCT). Increasing the LWIR (blue) absorber thickness is the common strategy used in dual–band photodetectors in order to reduce spectral cross–talk. As shown in Fig. 4, the residual LWIR response (λ < 0038 μm) of the red channel is reduced because of using a 4 μm–thick blue absorption region in the photodetector. Further improvement can be obtained by using even thicker blue absorption region.

**Figure 5:**
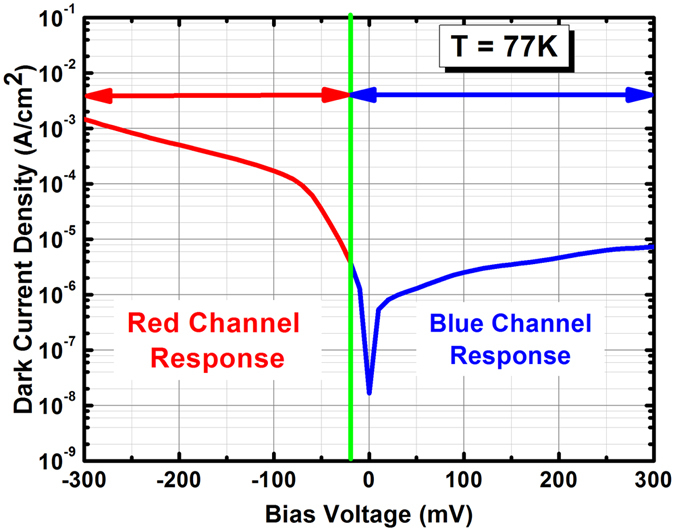
Dark current density vs. applied bias voltage of the dual–band LWIR/VLWIR photodetector at 77 K. The blue and red curves represent the operational bias regimes in which the device acts like a blue and red photodetector, respectively.

After optical characterization, the photodetectors were covered with a 77 K zero–field–of–view cold–shield to allow assessment of the electrical performance (see Fig. 5). At 77 K, the dark current density of the photodetector at +40 and −100 mV of applied bias are 1.13 × 10^−6^ and 1.7 × 10^−4^ A/cm^2^, respectively, which correspond to the bias voltages necessary to saturate the quantum efficiency spectrum of the blue and red channels. The differential resistance × area product (R × A) at +40 and −100 mV bias are 62938 and 407 Ω·cm^2^, respectively, at 77 K.

**Figure 6:**
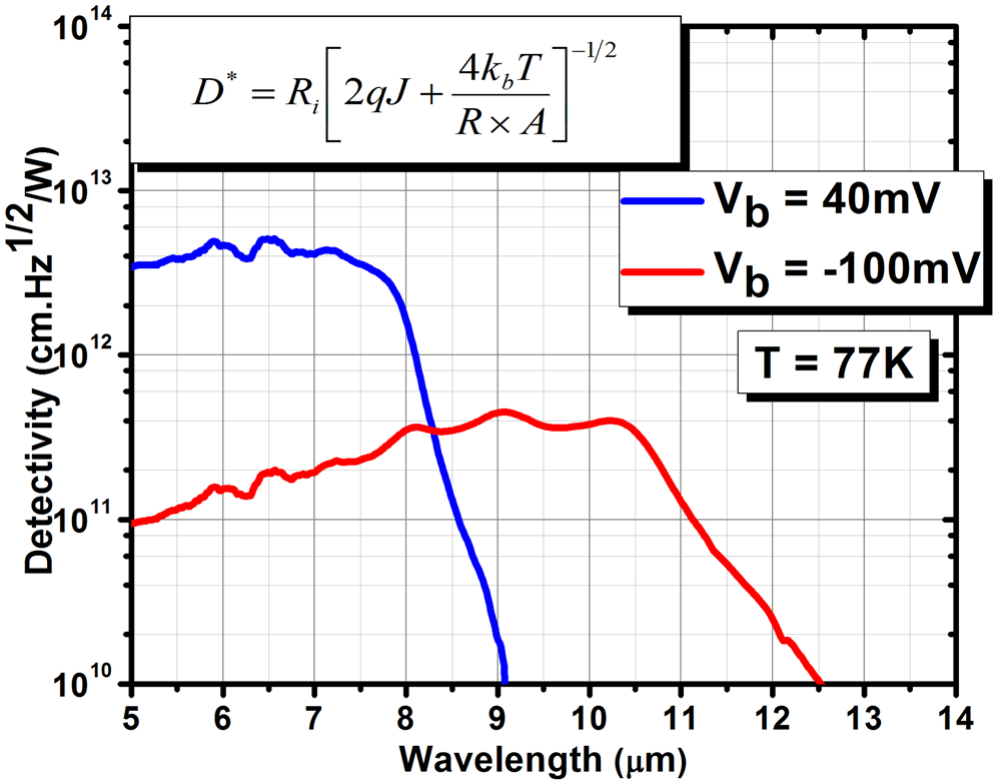
Saturated shot noise–limited specific detectivity spectrum of the blue and red channels at +40 and −100 mV applied bias voltage (V_b_), respectively, in front-side illumination configuration without any anti-reflection coating. This specific detectivity is calculated using the equation in the inset, where *R*_*i*_ is the device responsivity, *J* is the dark current density, *R* × *A* is the differential resistance × area product, *k*_*b*_ is the Boltzmann constant, and *T* is the operating temperature.

After complete optical and electrical characterization, the specific detectivity (D*) was calculated for both the blue and red channels. The blue channel exhibits a saturated dark current shot noise limited specific detectivity of 5.1 × 10^12^ cm·$\sqrt{Hz}$/W (at a peak responsivity of 6.45 μm) under +40 mV of applied bias, and the red channel’s specific detectivity was 4.5 × 10^11^ cm·$\sqrt{Hz}$/W (at a peak responsivity of 9 μm) under −100 mV of applied bias (see Fig. 6). In Fig. 6, both blue and red channels attain specific detectivity values of >1 × 10^11^ cm·$\sqrt{Hz}$/W over a broad range of wavelengths and stay above the background–limited infrared performance (BLIP) line (<5 × 10^10^ cm·$\sqrt{Hz}$/W for a fully immersed 300 K background with a 2π–field–of–view) until they approach to their cut–off wavelengths. In addition, the specific detectivity spectrums of both channels stay almost constant over a broad range of wavelengths which makes this bias–selectable dual–band LWIR photodetector an ideal choice for two–color infrared imaging applications (see Fig. 6).

## Discussion and Conclusion

In short, we have reported the design, growth, and characterization of a high–performance bias–selectable dual–band LWIR/VLWIR nBn photodetectors based on InAs/InAs_1−x_Sb_x_/AlAs_1−x_Sb_x_ T2SLs. The electron–barrier was found using a novel saw–tooth superlattice design we developed to allow a relatively high conduction band energy and to have near–zero discontinuity in valence band with respect to both absorption regions. At 77 K and +40 mV of applied bias, the blue channel achieves a saturated quantum efficiency of 65% under front–side illumination and without any AR coating, and the device exhibits a dark current density of 1.13 × 10^−6^ A/cm^2^ leading to a specific detectivity of 5.1 × 10^12^ cm·$\sqrt{Hz}$/W at 77 K. The red channel has a saturated QE of 50% and a dark current density of 1.7 × 10^−4^ A/cm^2^ at −100 mV, leading to a specific detectivity of 4.5 × 10^11^ cm·$\sqrt{Hz}$/W which stays constant over a broad range of wavelengths.

This technology is ideal for the realization of two-color LWIR/VLWIR focal plane arrays since it combines both LWIR and VLWIR detection into a single photodetector that can be tuned to either wavelength by changing the applied bias +40/−100 mV.

This work has made it possible for the InAs/InAs_1−x_Sb_x_/AlAs_1−x_Sb_x_ type–II superlattices to become a strong candidate for making multi–spectral infrared imagers in the LWIR and VLWIR regions and a possible replacement for current state–of–the–art technologies like InAs/GaSb/AlSb T2SLs.

## Methods

### Epitaxial Growth and photodetector fabrication

The photodetector material was grown using the Molecular Beam Epitaxy (MBE) equipped with group III SUMO® cells and group V valved crackers on tellurium *n* ^+^–doped (001) GaSb substrates. Silicon was introduced in InAs as the *n*–type dopant. The Reflection high energy electron diffraction (RHEED) method was used as the *in situ* tool to monitor the epitaxial growth process.

After structural characterizing the grown material and confirming to be of high quality, the material will be processed into single–element photodetectors for characterizing its optical and electrical performance. For the mesa isolation, material will be first covered with photoresist (PR) by spincoating. By controlling the spinning speed, the thickness of the PR can be adjusted. Usually the deeper the etching depth is, the thicker the PR is requested. Moreover, for different etching mechanisms, the type of PR can be changed. After spinning, the PR needs to be soft baked to make it become hard enough for the lithography. Since PR is ultra–violet (UV) sensitive material, its chemical property changes when it exposes to the UV light. The exposure area becomes soft for the positive PR but hard for the negative PR. To define the shape of the mesa, a photo mask is placed between the PR and the UV light source. After development, the pattern of the mask will be transferred on the PR.

The pattern of PR was be transferred onto the material to form single–element photodetector by a combination of physical and chemical etch processes; inductively coupled plasma–reactive ion etching (ICP–RIE) (Oxford PlasmaLab System 100) and citric–acid based solution were used for dry and wet etching, respectively. In the physical process, Argon ion plasma is used to bombard the material and break the chemical bonds in the material and the etching is anisotropic and directional. In the chemical process, the BCl_3_ plasma is used to react with the material, which can accelerate the etching process. The drying process can has very high uniformity, reduce the undercut, and improve the sidewall verticality. However, since the dry etch residue is left on mesa sidewall, which degrades the overall performance, an isotropic citric–acid based chemical wet etching is applied after ICP–RIE dry etching to remove the residue and regenerate a smoother sidewall.

After etching, cleaning procedure was applied to remove the PR residual and byproducts from the etching step that may create band bending in the vicinity of the sidewalls. AZ KWIK strip remover at high temperatures (100 °C) was used to clean the PR after each photolithography, and then complementary cleaning was applied using high purity solvents (acetone, methanol, and propanol). This clean procedure is very important because this residual and byproduct can easily cause surface leakage current, which degrades the photodetector’s electrical performance by orders of magnitude.

Once the cleaning is completed, the top and bottom metal contacts deposition is performed by electron beam metal evaporation, following by lift–off processing. The PR profile in this step requires a negative profile, which means the bottom of the PR will be developed faster than the top. This allows for the metal covering the unwanted area to be removed. Care selection of metal for top and bottom contacts is required because an ohmic contact must be formed to prevent potential barriers between the semiconductor and metal junction. A CHS electron beam evaporator was used to deposit Ohmic metal contact (Ti/Au stack) for the top and bottom contacts of the photodetectors. During the metallization, to ensure uniform thickness of metal layers, the evaporation rate was controlled (<2 Å/sec) and the deposition thickness was monitored in real–time by a quartz crystal. After lift–off, the same cleaning procedure is performed as before. Then, the sample was left unpassivated but special attention was paid in order to minimize the surface leakage during all fabrication procedure.

### Photodetector electro–optical testing

After fabrication of single–element photodetectors, the sample was mounted onto a 68–pin leadless ceramic chip carrier (LCCC) by indium for electro–optical characterization. The connections from the top and bottom contacts of the photodetectors were bonded to the bond pads of the LCCC. The bonds were made with a resistance–heated thermo compression wire bonder. After wire–bonding, the sample was loaded into a Jenis STVP–100 liquid helium cryostat. The devices were connected to different testing equipments using switching matrix for various type of measurement. The optical response measurement was divided into relative spectral response and blackbody integrated response sub–measurements. The photodetector spectral response was measured using a Bruker IFS 66v/S Fourier transform infrared spectrometer (FTIR) and the responsivity and QE were measured with a calibrated blackbody source at 1000 °C.
